# Isolated Torsion of the Fallopian Tube: A Novel Presentation

**DOI:** 10.7759/cureus.83381

**Published:** 2025-05-03

**Authors:** Vaia Sarli, Pavlos Machairoudias, Maria-Gesthimani Housmekeridou, Konstantinos Louis, Charikleia Papageorgiou, Dimos Sioutis, Periklis Panagopoulos, Nikolaos Machairiotis

**Affiliations:** 1 Third Department of Obstetrics and Gynecology, Attiko University Hospital, National and Kapodistrian University of Athens School of Medicine, Athens, GRC

**Keywords:** acute pelvic pain, fertility preservation, isolated salpinx torsion, laparoscopy, salpingectomy

## Abstract

Isolated fallopian tube torsion (IFTT) is a rare cause of acute pelvic pain that is often misdiagnosed due to its non-specific clinical findings. Early diagnosis and operative intervention are vital for the preservation of fertility. We report two cases of IFTT.

The first case is of a 16-year-old female with a seven-day history of right lower abdominal pain and fevers. No flow was noted on Doppler studies of the right adnexal mass on ultrasound. Laparoscopy showed torsion of the fallopian tube, and a salpingectomy was performed. The second case was in a 42-year-old woman with a two-day history of lower abdominal pain, nausea, and vomiting. Radiology revealed a right fallopian tube cystic mass with decreased perfusion. Histopathology of the specimen showed ischemic changes, and a laparoscopic salpingectomy was performed. The postoperative course of both patients was uneventful.

IFTT is a rare but clinically significant differential diagnosis of acute pelvic pain. A high index of suspicion, early imaging, and laparoscopic definitive management aid in optimizing outcomes and preserving fertility in these patients.

## Introduction

Isolated fallopian tube torsion (IFTT) is very uncommon, and such conditions can be classified into two groups: intrinsic causes, such as congenital malformations, hydrosalpinx, pelvic inflammatory disease (PID), and tubal ligation; and extrinsic causes, such as adhesions, endometriosis, tumors, ectopic pregnancy, and trauma. Laparoscopy is the gold standard for diagnosis and management. IFTT is frequently underdiagnosed due to poor clinical presentations and nonspecific imaging findings. Early detection and urgent surgical intervention are important for early management and for preserving fertility and avoiding salpingectomy [1-5].

## Case presentation

**Figure 1:**
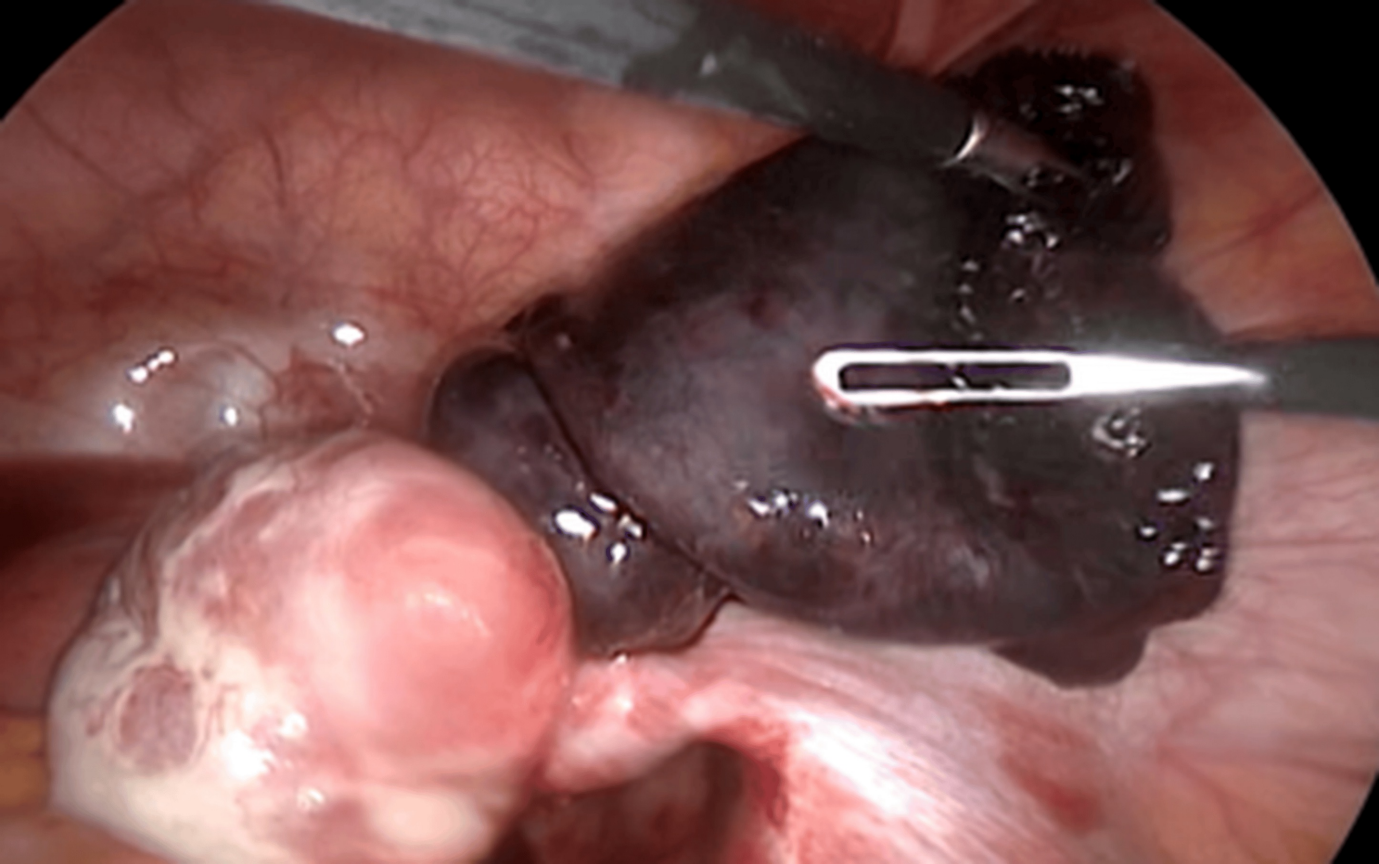
Necrotic, torsioned right fallopian tube with normal ovary (laparoscopic view, Case 1).

**Figure 2:**
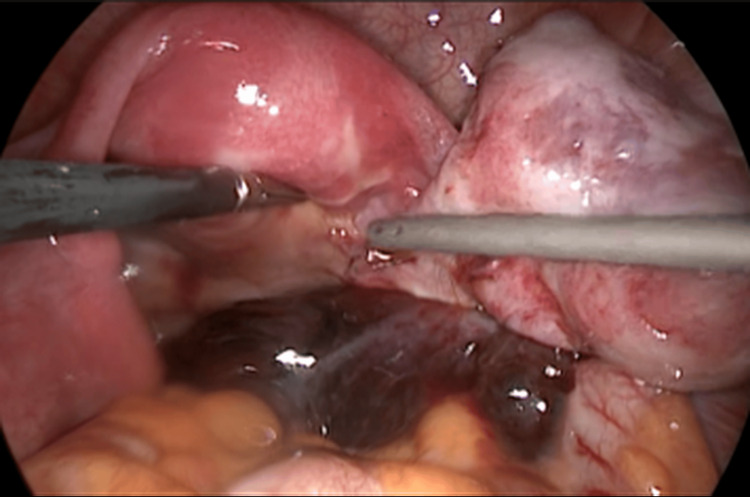
Torsioned right fallopian tube and normal-appearing ovary (Case 1).

Two cases of isolated fallopian tube torsion are presented below. The first case was a 16-year-old sexually inactive female with a 7-day history of right lower abdominal pain and fever (37.8 °C). Laboratory data showed leukocytosis (WBC: 16,500). Pelvic ultrasound revealed a right adnexal mass (13 × 10 mm) without Doppler flow. She underwent emergency laparoscopy, which revealed a necrotic, torsioned right fallopian tube and a normal-appearing ovary. She underwent a laparoscopic right salpingectomy. Histopathological examination demonstrated hematosalpinx causing ischemic changes in the fallopian tube (Figures 1-2, Table 1).

**Table 1:** Laboratory findings in patients with isolated fallopian tube torsion (Cases 1 and 2).

Lab Test	Case 1 (16-year-old)	Case 2 (42-year-old)	Normal Range
WBC	16,500/μL	Normal	4,000-11,000/μL
Temperature	37.8 °C	Normal	36.1-37.2 °C
Pelvic Ultrasound	Right adnexal mass (13 × 10 mm)	Right fallopian tube cystic mass (8 cm)	N/A
CT Scan/Imaging	No Doppler flow	Diminished perfusion	N/A

**Figure 3:**
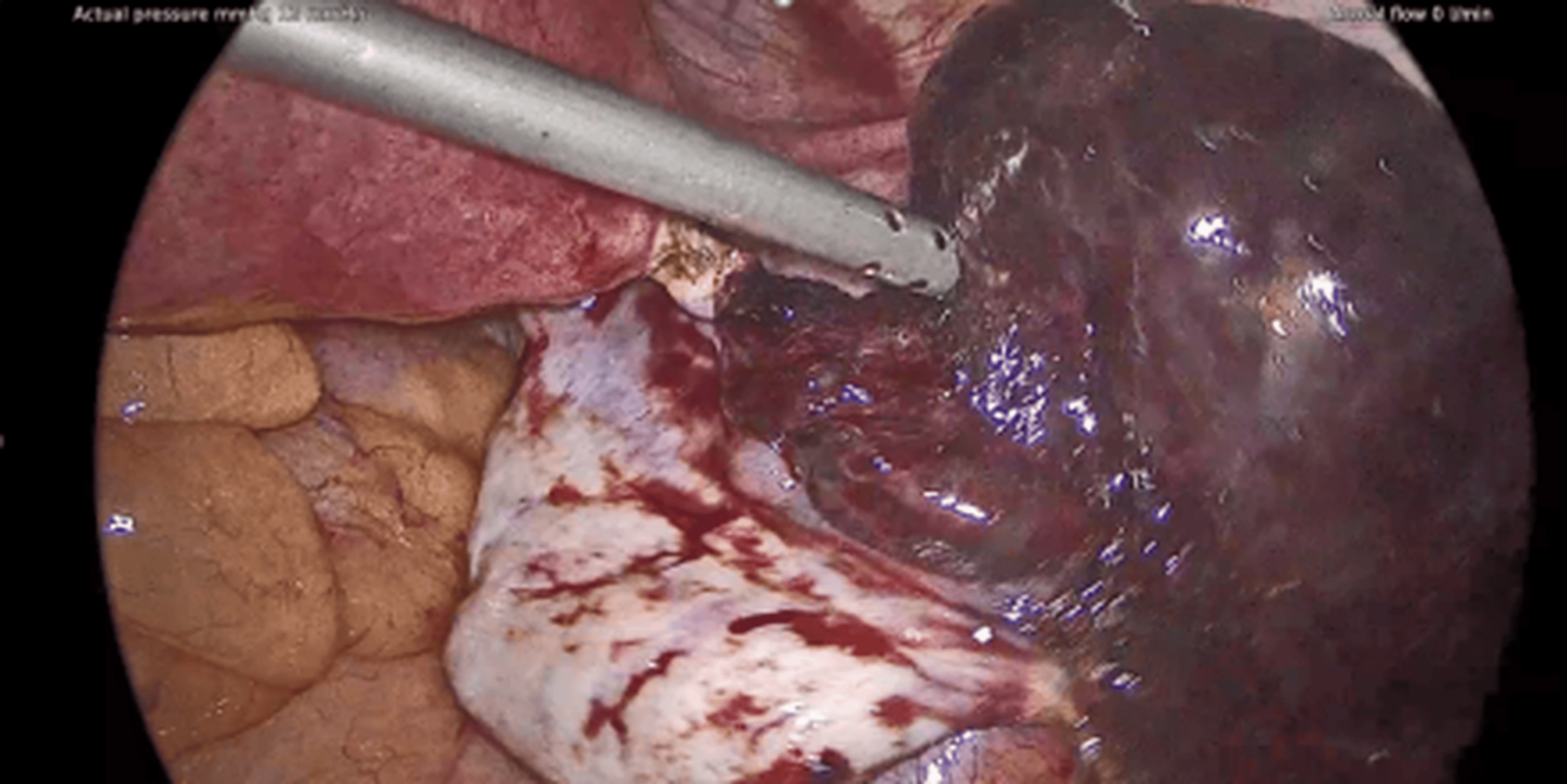
42-year-old with isolated fallopian tube torsion and normal ovarian appearance (Case 2).

**Figure 4:**
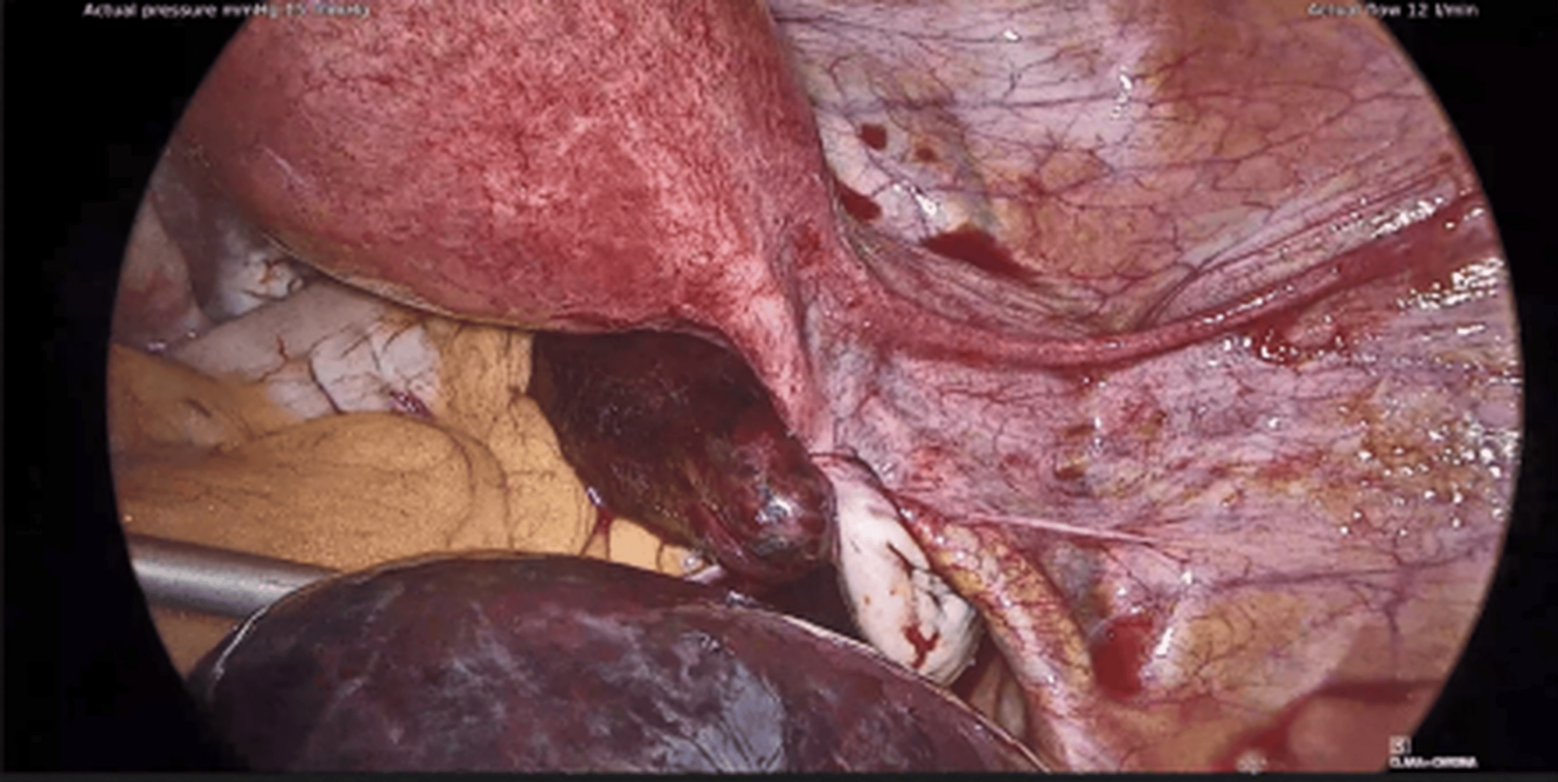
Right isolated fallopian tube torsion (laparoscopic view, Case 2).

The second case was a 42-year-old woman evaluated for a two-day history of lower abdominal pain, nausea, and vomiting. She was afebrile with a normal WBC count. Imaging (CT and transvaginal ultrasound) showed a right fallopian tube cystic mass (8 cm) with diminished perfusion. The uterus and ovaries appeared normal. A laparoscopic right salpingectomy for suspected torsion of the fallopian tube was performed. Intraoperatively, the right tube was found to be dark red and twisted. Histological examination confirmed ischemic changes. She had an unremarkable postoperative course and was discharged on the first postoperative day (Figures 3-4).

## Discussion

IFTT continues to be a diagnostic challenge due to its nonspecific symptoms. It should be considered among the etiologies of acute pelvic pain, especially in cases where ovarian torsion is suspected but not confirmed. Imaging studies such as ultrasound and CT scans are important, but laparoscopy is required for definitive diagnosis and treatment. Management primarily aims to preserve fertility whenever possible, but delayed diagnosis often leads to salpingectomy. These cases highlight the importance of increased clinical awareness to reduce diagnostic delay and improve reproductive outcomes [4,5].

The differential diagnosis for IFTT is characterized by nonspecific symptomatology that can mimic various gynecological and non-gynecological conditions. For instance, ovarian torsion, commonly associated with sudden onset of severe pelvic pain, often accompanied by nausea and vomiting, is one differential diagnosis; however, it involves the ovary, whereas ectopic pregnancy involves the fallopian tube. An ectopic pregnancy may also be suspected in the presence of a positive pregnancy test along with lower abdominal pain and vaginal bleeding [2].

Hemorrhagic ovarian cysts [2] can cause acute pelvic pain secondary to cyst rupture and hemoperitoneum. Appendicitis often presents with tenderness in the right lower quadrant, accompanied by fever and elevated inflammatory markers [4]. PID should also be considered, typically causing dull pelvic pain in combination with fever, cervical motion tenderness, and, in some cases, abnormal vaginal discharge [5]. Lastly, endometriosis-related pelvic pain should be included in the differential diagnosis. While typically presenting as chronic pelvic pain, it may present acutely in cases of endometriomas or deep infiltrating disease [5].

## Conclusions

IFTT is a rare but important cause of acute pelvic pain. Although imaging modalities have improved over the years, early clinical suspicion remains vital for timely diagnosis and management. Laparoscopy is the most common diagnostic and therapeutic method. Considering that delayed diagnosis is common, but tubal preservation is optimal, salpingectomy is often required. These cases highlight the need for early recognition to optimize fertility prospects.
